# Performance of the Abbott SARS-CoV-2 IgG serological assay in South African 2 patients

**DOI:** 10.1371/journal.pone.0262442

**Published:** 2022-02-04

**Authors:** Sarika Jugwanth, Maemu P. Gededzha, Nakampe Mampeule, Nontobeko Zwane, Anura David, Wendy A. Burgers, Jonathan M. Blackburn, Jurette S. Grove, Jaya A. George, Ian Sanne, Lesley Scott, Wendy Stevens, Elizabeth S. Mayne

**Affiliations:** 1 Department of Immunology, Faculty of Health Sciences, University of the Witwatersrand, Johannesburg, South Africa; 2 National Health Laboratory Services, Johannesburg, South Africa; 3 Department of Molecular Medicine and Haematology, School of Pathology, Faculty of Health Sciences, University of Witwatersrand, Johannesburg, South Africa; 4 Division of Medical Virology, Department of Pathology, Faculty of Health Sciences, University of Cape Town, Cape Town, South Africa; 5 Wellcome Centre for Infectious Diseases Research in Africa, Cape Town, South Africa; 6 Institute of Infectious Disease and Molecular Medicine, University of Cape Town, Cape Town, South Africa; 7 Divisions of Chemical and System Biology, Department of Integrative Biomedical Sciences, Faculty of Health Sciences, University of Cape Town, Cape Town, South Africa; 8 Department of Chemical Pathology, Faculty of Health Sciences, University of the Witwatersrand, Johannesburg, South Africa; 9 Clinical HIV Research Unit, Department of Medicine, Faculty of Health Sciences, University of the Witwatersrand, Johannesburg, South Africa; Academia Sinica, TAIWAN

## Abstract

In late December 2019, pneumonia cases of unknown origin were reported in Wuhan, China. This virus was named SARS-CoV2 and the clinical syndrome was named coronavirus disease 19 (COVID-19). South Africa, despite strict and early lockdown has the highest infection rate in Africa. A key component of South Africa’s response to SARSCoV2 was the rapid scale-up of diagnostic testing. The Abbott SARS-CoV2 assay detects IgG antibodies against the Nucleocapsid (N) protein of the SARS-CoV2 virus. This study undertook to validate and evaluate performance criteria of the Abbott assay and to establish whether this assay would show clinical utility in our population. Positive patients (n = 391) and negative controls (n = 139) were included. The Architect-i and Alinity-i systems were analyzers that were used to perform the SARS-CoV-2 IgG assay. In-house ELISA was incorporated into the study as a confirmatory serology test. A total of number of 530 participants was tested, 87% were symptomatic with infection and 13% were asymptomatic. When compared to RT-qPCR, the sensitivity of Architect and Alinity SARS-CoV2 assays was 69.5% and 64.8%, respectively. Specificity for Architect and Alinity assays was 95% and 90.3%, respectively. The Abbott assay was also compared to in house ELISA assay, with sensitivity for the Architect and Alinity assays of 94.7% and 92.5%, respectively. Specificity for Abbott Alinity assays was 91.7% higher than Abbott Architect 88.1%. Based on the current findings testing of IgG after 14 days is recommended in South Africa and supports other studies performed around the world.

## Introduction

In late December 2019, pneumonia cases of unknown origin were reported in Wuhan, the Hubei province of China [1]. The virus was identified as a novel betacoronavirus, the seventh coronavirus identified to cause infections in humans [2]. Due to its genetic similarity to SARS-CoV, this virus was named SARS-CoV-2 [3] and the clinical syndrome was named coronavirus disease 19 (COVID-19) by the WHO [1, 4, 5]. Although the animal source is still unconfirmed, this novel virus is also believed to be of zoonotic origin, introduced through the Chinese wet market [6]. Similar to related viruses SARS-CoV and MERS-CoV, COVID-19 manifests as a wide spectrum of symptoms from mild upper respiratory tract symptoms to severe disease [4]. Mortality has been linked to increased age and is seen in those with comorbid illnesses [2, 3, 7, 8]. An international public health concern was raised [1] and a global pandemic was declared by the WHO [2]. As of July 28^th^ 2021, about 194 million individuals were infected with more four million deaths were reported worldwide [9]. The course of the pandemic in Africa was of concern because of weaknesses in health systems in some African countries and the high burden of both non-communicable and communicable disease [10]. South Africa, despite its strict and early lockdown, is the country in Africa with the highest infection rate. This lockdown was one of the most stringent worldwide and aimed to protect the healthcare services and to protect vulnerable populations, especially those with Human Immunodeficiency Virus (HIV) infection and tuberculosis (TB) [2, 10–12].

A key component of the South African response to the pandemic was the rapid scale-up of diagnostic testing. Reverse Transcription-Polymerase Chain Reaction, performed on nasopharyngeal swabs, is the current gold standard method for the acute diagnosis of COVID-19, although global demand for kits is high with consequent constraints on turnaround times and in some cases, limitations on testing [13, 14]. Testing in South Africa was, at times, limited to vulnerable and symptomatic populations only [15]. On the 21^st^ March the WHO published recommendations on the use of serological testing as part of research, surveillance, monitoring response and recovery studies of COVID-19 infected individuals [13, 14, 16].

The Abbott SARS-CoV-2 IgG assay (Abbott Diagnostics, Abbott Park, Illinois, United States) is a high-throughput chemiluminescent assay which is designed to detect IgG antibodies against the Nucleocapsid (N) protein of the SARS-CoV-2. The advantage of detecting IgG is its persistence after established infection, allowing retrospective diagnosis in convalescent individuals. N protein is antigenically highly immunodominant and antibodies have, in some cases, correlated with levels of protective neutralizing antibodies [17]. The Abbott assay is designed to run on both Abbott Architect™ and Alinity™ analysers (Abbott Diagnostics, Abbott Park, Illinois, United States). High specificity and improved clinical sensitivity for the detection of IgG antibody from day 14 post symptom onset using the Abbott SARS-CoV-2 IgG assay was described in several studies [3, 8, 18–21]. Table 1 details a range of studies conducted worldwide, showing a cumulative sensitivity range of 38.8%-97.9%. The Immunology Laboratory at the National Health Laboratory Service is a tertiary referral facility servicing the Southern Johannesburg region. Early in the pandemic, this laboratory undertook to validate and evaluate performance criteria of the Abbott assay and to establish whether this assay would show clinical utility in our population.

**Table 1:** Cumulative review of Abbott Architect SARS IgG validation studies.

Study	Number of samples tested	Sensitivity	Specificity
[3] France	Patients:141	Time from onset of symptoms: Cumulative Sensitivity: 81.8% (95% CI: 74.7–87.3%)	Cumulative Specificity: 99.3% (CI 95%: 96.3–99.9%)
	Controls:152		
[18], Singapore	Patients: 170	Time from onset of symptoms: Cumulative sensitivity 38.8% (95%CI: 31.8–46.3%)	Cumulative specificity: 100%
	Controls: 163		
[19], USA	Patients: 97	Time from onset of symptoms Cumulative sensitivity: 97.9% Time from RT-qPCR positivity Overall sensitivity: 97.9%	Cumulative specificity: 99.6%
	Controls: 215		
[20], Denmark	Patients: 150	Time from onset of symptoms Cumulative sensitivity: 90% (95%CI: 84.2–93.8%)	Cumulative specificity: 99.5% (95% CI: 98.5%-99.8%)
	Controls: >586		
[8], Italy	Patients: 140	Cumulative sensitivity: 72% (95% CI: 64.3%-79.6%)	Cumulative specificity: 100%
	Controls: 37		

## Methods

### Sample and data collection

This study was approved by the Human Research Ethics Committee of the University of Witwatersrand (M200468) for voluntary participation and (M1911201) for the use of archived pre-pandemic samples in the study as negative controls. Voluntary participants over 18 years were invited to participate in a fourway validation of serological and rapid point-of-care testing for SARS-CoV2 in South Africa. Research information and the purpose were explained to participants first and thereafter they were asked to sign the written informed consent form. Only participants who provided written informed consent included in the study. Participants were requested to complete a questionnaire regarding their demographic information, symptomatology and to declare any comorbidities, medications, and travel history (S1 Appendix). Informed consent was obtained to collect up to four tubes of EDTA venous blood and two to six tubes of venous blood in serum separator tubes. Participant samples were de-identified and assigned a unique study identifier. Research data for samples that had no information that could be used to trace back to a patient, was not released or published.

#### Participants

Participants who tested positive (n = 391) by Quantitative reverse transcription PCR (RT-qPCR) were used as positive controls.

Positive controls were stratified into the following groups according to the number of days post-PCR (in asymptomatic individuals) or post-symptom onset.

1. Timepoint 1: Day 0–7 post positive presentation (n = 77)
2. Timepoint 2: Day 8–14 post positive PCR result (n = 69)
3. Timepoint 3: Day 15–21 post positive PCR result (n = 48)
4. Timepoint 4: Day 22–30 post positive PCR result (n = 48)
5. Timepoint 5: Day 31–40 post positive PCR result (n = 35)
6. Timepoint 6: Day 41–50 post positive PCR result (n = 29)
7. Timepoint 7: >51 days post positive PCR result (n = 85)

Positive controls were further stratified into asymptomatic, mildly symptomatic (upper respiratory tract infections), moderately symptomatic (lower respiratory tract infections, high fever or severe gastrointestinal symptoms) and severely symptomatic (admitted into health care facility to treat disease) using the clinical questionnaires.

*Negative controls*. Negative controls were identified for testing from 4 different sources (n = 139):

1. Well-characterised stored serum samples which were stored in a biorepository prior to February 2020
2. Contacts of infected patients who tested negative by RT-qPCR on two separate occasions and had no identifiable antibodies on in-house serology testing
3. Stored serum samples from patients with viral pneumonia prior to February 2020
4. Stored serum samples from individuals attending clinics for connective tissue disorders that previously tested positive for antinuclear antibodies (ANA) by Indirect Immunofluorescence Assay (IFA) with specific antibodies against SSA (Ro), SSB (La), Smith and/or RNP were included in this study to test for cross reactivity.

### Sample preparation

Samples were maintained at ambient temperatures during transportation. Samples were received at the Immunology laboratory at the National Health Laboratory Service, Braamfontein campus within 4 hours of sample collection and processed within 24 hours.

Samples were centrifuged utilising either the Rotina 420R or 460R centrifuges (Andreas Hettich GmbH & Co. KG, Tuttlingen, Germany) at 3500 rpm for serum tubes and 800 rpm for EDTA tubes. Serum and plasma were stored as 200ul aliquots frozen at -80°C and freeze-thaw cycles were limited to one. Once thawed, the samples were run immediately.

### Abbott Architect and Alinity SARS CoV2 IgG

Architect™ and Alinity™ analysers are electronic chemiluminescent microparticle immunoassay (CMIA) analysers that were used to perform the SARS-CoV-2 IgG assay (CMIA) for detection of the antibodies to nucleocapsid protein of SARS-CoV-2 in human plasma and/ or serum samples. The chemiluminescent reaction is measured as a relative light units (RLU) and expressed as a calculated index (S/C). Results greater than 1.4 are interpreted as positive. The procedure was performed according to the manufacturer’s instructions.

### Enzyme-Linked Immunosorbent Assay (ELISA)

In-house ELISAs were performed on a subgroup of the SARS-CoV-2 samples (n = 197) as a confirmatory serological tests as detailed below:

ELISA assays was used for the detection IgG binding to the S1 domain of the spike protein, which was modified from FDA-approved protocol [21, 22], to clone, express and purify recombinant S1 from the Nicotiana benthamiana plants.

96-well plates (Nunc MaxiSorp, Thermo Fisher, Waltham, Massachusetts, United States) coated with purified recombinant S1 were incubated for 2 hours at room temperature with individual patient samples diluted, 1:50 in PBS. Goat anti-human IgG (Fc-specific) peroxidase conjugate was added for signal detection [21]. The mean plus 2 standard deviations was used as a threshold for a true positive signal, as determined using data from pre-pandemic controls (n = 58).

### Statistical analysis

The mean, standard deviation, median and interquartile range were calculated where relevant. STATA 14 (StataCorp LLC, Texas, and USA) software for statistic and data science was utilized for measuring sensitivity and specificity against true positives (defined against RT-qPCR) and negatives, and expressed as a percentage. Assay performance against RT-qPCR and in-house serology methods were calculated as an aggregated sensitivity and specificity and then disaggregated according to days post diagnosis and by symptom. Values were expressed as a percentage. Intrarun precision was measured by running 5 replicate samples inclusive of high and low values and the same 5 replicates were run over 5 days for interrun precision. These were reported as a % coefficient of variation.

## Results

### Participant characteristics

A total of number of 530 participants were tested. Of these, 356 infected participants (67%) had recorded the presence or absence of symptoms on the questionnaires. In total, 87% had symptomatic infection and 13% were asymptomatic (Table 2).

**Table 2:** Participant characteristics.

	Positive controls (n = 391)	Negative controls (n = 139)
Age in years: Median (range)	41 (20–82)	43 (22–74)
Male %	43%	43%
Female %	57%	57%
Ethnicity		
African %	33%	18%
Caucasian %	43%	77%
Indian %	14%	5%
Mixed race	10%	0%
**Symptomatology**		
Asymptomatic n, %	48, 13.5%	
Mildly symptomatic n, %	30, 8.4%	
Moderately symptomatic n, %	169, 47.5%	
Severe symptomatic n, %	109, 30.6%	
Days post presentation n, %		
0–7 days	77, 19.7%	
8–14 days	69, 17.7%	
15–21 days	48, 12.3%	
22–30 days	48, 12.3%	
31–40 days	35, 8.9%	
41–50 days	29, 7.4%	
>51 days	85, 21.7%	

### Assay performance against RT-qPCR

The cumulative sensitivity of both the Abbott SARS-CoV2 Architect and Abbott SARS-CoV2 Alinity assays when compared to RT-qPCR was 69.5% (95% CI: 64.7% 74.1%) and 64.8% (95% CI: 59.4%-69.9%), respectively. The sensitivity for both assays were highest at 31–40 days post presentation, and lowest at timepoint less than 7 days (Table 3). The diagnostic specificity was for the Abbott SARS-CoV2 Architect and Abbott SARS-CoV2 Alinity assays was 95% (95% CI: 89.9%-98%) and 90.3% (95% CI: 82.9%95.2%) out of a total of 526 and 435 samples tested, respectively (Table 3). In addition, both assays tested the same 25 positive ANA samples and did not demonstrate any cross-reactivity to any of the autoimmune disorders.

**Table 3:** Sensitivity of each assay compared with RT-qPCR.

		Abbott SARS-CoV2 Architect Assay (n = 526)	Abbott SARS-CoV2 Alinity Assay (n = 435^a^)
Sensitivity %	Cumulative sensitivity	69.5% (95% CI: 64.7% -74.1%)	64.8% (95% CI: 59.4%-69.9%)
	0–7 days	43/77, 55.8%	37/69, 53.6%
	8–14	46/68, 67.6%	34/57, 59.6%
	15–21	36/47, 76.6%	32/43, 74.4%
	22–30	26/46, 56.5%	21/41, 51.2%
	31–40	30/35, 85.7%	22/28, 78.6%
	41–50	21/29, 72,4%	17/22, 77.2%
	>51	56/71, 78.8%	41/58, 70.7%
Cumulative specificity %		95% (95% CI: 89.9%-98%)	90.3% (95% CI: 82.9%-95.2%)

^a^Due to sample availability, the number of samples used for the Abbott SARS-CoV2 Alinity Assay differs from that used for the Abbott SARS-CoV2 Architect Assay.

Table 4 below compares assay performance to RT-qPCR by reported clinical presentation. The sensitivity based on clinical presentation was highest in severely symptomatic patients when measured more than 14 days for both assays.

**Table 4:** Assay performance compared with RT-qPCR delineated by reported clinical presentation.

	Abbott SARS-CoV2 Architect Assay		Abbott SARS-CoV2 Alinity Assay	
Clinical presentation	0–14 days (n = 120)	>14 days (n = 230)	0–14 days (n = 108)	>14 days (n = 192)
Asymptomatic	41.6%	41.6%	36.4%	45.5%
Mild	33.3%	57.1%	33.3%	47.1%
Moderate	47.2%	81.2%	39.4%	73.3%
Severe	74.6%	90%	72.7%	83.3%

### Assay performance against in-house ELISA

The sensitivity of the Abbott SARS-CoV2 Architect and Abbott SARS-CoV2 Alinity assays was calculated on a total number 197 samples and 191 samples, respectively, compared to in-house ELISA that measured spike IgG (Table 5). A good overall sensitivity was obtained for the Abbott SARS-CoV2 Architect and Abbott SARS-CoV2 Alinity assays, of 94.7% (95% CI: 88.8% -98%) and 92.5% (95% CI: 85.8%-96.7%), respectively. The sensitivity for both assays were highest at time-points 31–40 days and lowest at timepoints 8–14 days. The diagnostic specificity for the Abbott Alinity assays was 91.7% (95% CI: 83.6%-96.6%) and higher than the Abbott SARS-CoV2 Architect assay 88.1% (95% CI: 79.2%-94.1%) (Table 5).

**Table 5:** Abbott anti-SARS-CoV-2 Architect and Alinity assays sensitivity of each assay compared with in-house ELISA.

		Abbott SARS-CoV2 Assay Architect (n = 197)	Abbott SARS-CoV2 Assay Alinity (n = 191^a^)
Sensitivity %	Cumulative sensitivity	94.7% (95% CI: 88.8%-98%)	92.5% (95% CI: 85.8%-96.7%)
	0–7 days	13/14, 92.8%	13/14, 92.9%
	8–14	11/12, 91.7%	7/8, 87.5%
	15–21	12/14, 85.7%	12/14, 85.7%
	22–30	10/12, 83.3%	10/13, 76.9%
	31–40	12/12, 100%	12/12, 100%
	41–50	14/14, 100%	14/14, 100%
	>51	32/32, 100%	29/30, 96.6%
Cumulative specificity %		88.1% (95% CI: 79.2%-94.1%)	91.7% (95% CI: 83.6%-96.6%)

^a^Because of sample availability, the number of samples used for the Abbott SARS-CoV2 Alinity Assay differs from that used for the Abbott SARS-CoV2 Architect Assay.

The sensitivity of clinical presentation was highest for moderate symptomatic patients post 14 days and was highest for severely symptomatic patient for the Abbott SARSCoV2 Architect assay 0–14 days (Table 6).

**Table 6:** Abbott anti-SARS-CoV-2 Architect and Alinity assays performance compared with in-house Elisa reported by clinical presentation (severity).

	Abbott SARS-CoV2 Architect Assay		Abbott SARS-CoV2 Alinity Assay	
Clinical presentation	0–14 days (n = 23)	>14 days (n = 85)	0–14 days (n = 21)	>14 days (n = 82)
Asymptomatic	NA^a^	90.9%	NA^a^	90.9%
Mild	NA^a^	85.7%	NA^a^	85.7%
Moderate	80%	94.4%	88.8%	94.3%
Severe	100%	92.3%	88.8%	90.9%

NA^a^- not available–Denotes that the number of patients were too small to give a true reflection of assay performance by clinical presentation compared to in-house Elisa

### Composite precision

The control material had a coefficient variation (CV) of 20.5% using the Abbott SARS-CoV2 Architect assay and coefficient variation (CV) of 16.3% using the Abbott SARS-CoV2 Alinity assay run on five samples comprising positive and negative controls and run in duplicate over 5 days.

## Discussion

In this study, we evaluated the performance the Abbott SARS-CoV-2 IgG Architect and the Abbott SARS-CoV-2 IgG Alinity assays using the Architect™ and Alinity™ analysers. The cumulative sensitivity of both assays was compared to RT-qPCR and was found to be similar to results by Meschi and colleagues [8]. When compared to inhouse ELISA assay that detects the spike protein antibodies, the cumulative sensitivity for both Abbott assays (N protein) were similar to the cumulative sensitivity in the study conducted by Harritshoej and colleagues [20]. Previous studies have reported that SARSCoV-2 IgG N protein antibodies seroconvert earlier than IgG spike protein antibodies [23]. Both the Abbott SARS-CoV-2 IgG Architect and the Abbott SARS-CoV-2 IgG Alinity assays had improved sensitivity 14 days after diagnosis which increased with severity of clinical presentation. These findings are similar to other studies that have shown that sensitivity of serological assays improves with severity of symptoms, as patients may seroconvert earlier, due to a stronger immune response than patients with milder symptoms [20, 24, 25]. Results from this study shows that the specificity was slightly lower than manufacturer specificity and other studies in samples collected prior to February 2020 and samples with autoimmune disease [8, 20, 26–28]. Suhandynata et al proposed a ‘two-platform approach’ to test groups with low SARS-CoV-2 prevalence [29]. Application of this approach will prove useful in utilising both the validated assays on the respective Abbott Architect™ and Alinity™ analyzers to eliminate the risk of testing groups with low SARS-CoV-2 prevalence in our South African setting. Furthermore, the Abbott Architect™ and the Alinity™ analysers are able to process approximately 70 and 170 test per hour respectively [30], making these high through-put platforms suitable for larger seroepidemiologic studies in South Africa. In addition, continuous loading of samples is an advantageous function for both analyzers for prioritizing urgent cases, unlike other ELISA platforms.

The Abbott assay detect antibodies to the N protein, which is vital for viral transcription, replication and assembly of the COVID-19 virus [30]. Studies show that the nucleocapsid gene is more stable and is conserved, in contrast to the spike proteins [31]. In addition, the N protein is expressed in abundance during an infection [31], indicating a possible higher immune response to the N protein, giving the Abbott assay an advantage over assays targeting antibodies to the spike protein. Suhandynata et al showed that positive serological results using the SARS CoV-2 IgG assay on the Abbott Architect™ analyser correlated with neutralization activity of IgG antibodies [29]. Furthermore, a study by Rodgers et al. showed that the Abbott serological assays are able to detect SARS CoV2 variants that emerged through this pandemic [32].

This study shows the ability of both the Abbott SARS-CoV2 Architect and the Abbott SARS-CoV2 Alinity assays to assess long-term immunity because of the high sensitivity 30 days post-infection. This assay is also useful for delayed complications of SARS-CoV2 infection including prolonged COVID-19 disease (also called “long” COVID) and multiinflammatory syndrome in children (MIS-C) which is thought to be associated with SARSCoV-2 virus through previous infections [33]. Both of these conditions appear to be linked to an abnormal antibody response as well as ongoing inflammation [34, 35].

One of the limitations of this study is that the Abbott IgM assay was not evaluated for clinical use and the sample variability in anti-SARS-CoV-2 IgM/IgG seroconversion was not assessed. In addition, we did not know the symptomatology of all the positive patients as well as potential errors that may have been made by individuals who self-reported their symptoms onset days on their questionnaires. Despite these limitations, and to our knowledge, this is the first paper that has demonstrated the diagnostic performance of these assays in an African setting or on large numbers of asymptomatic patients and patients at later timepoints post-infection. This provides important ancillary data to support the use of these tests in the public health setting as well as in complicated COVID cases.

Quantitative reverse transcription PCR is the gold standard for acute diagnosis of SARSCOV-2, but has limitations that may impact test performance. These limitations include preanalytical issues (including specimen integrity and viral load) and in addition requires high levels of technical expertise.

In the real-world, the SARS-CoV-2 infection confirmed cases should be quarantined. For control the transmission infection occurred from the confirmed case, all possible contacted person (included asymptomatic-cases) should also be quarantined and screened for SARS-CoV-2 by RT-qPCR or viral antigen detection. The serological assay can used for the timing of 8–14 days or later post onset of infection. During the same period, the sensitivity of RT-qPCR is lower than the samples collected in the early stage of infection [36]. Epidemiological surveillance using serology testing is important as it has a shorter turn-around, is less expensive and less labour- intensive than RT-qPCR and is less invasive for the patients than collection of a nasopharyngeal swab. This study reports the diagnostic performance of the Abbott assay on RT-qPCR -confirmed SARS-CoV-2 infected patients.

In conclusion, the implementation of serological antibody testing was a national high priority and as a result, emergency validation protocols were written up and followed for which the data in this study was derived from. This study validates the clinical utilization of Abbott SARS-CoV-2 IgG assays with satisfactory overall sensitivity and excellent specificity performance. In addition, evaluating the suitability, ease of use and short turnaround times of the Abbott Architect™ and Alinity™ analysers as high throughput immunoassay analysers for handling high volumes of samples during this pandemic.

Based on the current findings, testing of IgG after 14 days is recommended in South Africa and supports other studies performed around the world. The utilization of these assays is recommended due to the ease of use for contact tracing, to improve understanding of the sero-epidemiology of COVID-19, such as in cases and aid with high volume testing that SA is currently faced with due to the new variant of COVID-19 virus.

## Supporting information

S1 Appendix Informed consent. (PDF)
